# Risk Factors for Aspiration Pneumonia in Older Adults

**DOI:** 10.1371/journal.pone.0140060

**Published:** 2015-10-07

**Authors:** Toshie Manabe, Shinji Teramoto, Nanako Tamiya, Jiro Okochi, Nobuyuki Hizawa

**Affiliations:** 1 Department of Pulmonary Medicine, Graduate School of Comprehensive Human Science, University of Tsukuba, Tsukuba, Ibaraki, Japan; 2 Department of Health Services Research, Faculty of Medicine, University of Tsukuba, Tsukuba Ibaraki, Japan; 3 Hitachinaka Medical Education and Research Center, University of Tsukuba, Hitachinaka, Ibaraki, Japan; 4 Tatsumanosato Geriatric Health Service Facility, Tatsuma, Daitou, Osaka, Japan; 5 Japanese Association of Geriatric Health Services Facilities, Tokyo, Japan; University of Dundee, UNITED KINGDOM

## Abstract

**Backgrounds:**

Aspiration pneumonia is a dominant form of community-acquired and healthcare-associated pneumonia, and a leading cause of death among ageing populations. However, the risk factors for developing aspiration pneumonia in older adults have not been fully evaluated. The purpose of the present study was to determine the risk factors for aspiration pneumonia among the elderly.

**Methodology and Principal Findings:**

We conducted an observational study using data from a nationwide survey of geriatric medical and nursing center in Japan. The study subjects included 9930 patients (median age: 86 years, women: 76%) who were divided into two groups: those who had experienced an episode of aspiration pneumonia in the previous 3 months and those who had not. Data on demographics, clinical status, activities of daily living (ADL), and major illnesses were compared between subjects with and without aspiration pneumonia. Two hundred and fifty-nine subjects (2.6% of the total sample) were in the aspiration pneumonia group. In the univariate analysis, older age was not found to be a risk factor for aspiration pneumonia, but the following were: sputum suctioning (odds ratio [OR] = 17.25, 95% confidence interval [CI]: 13.16–22.62, p < 0.001), daily oxygen therapy (OR = 8.29, 95% CI: 4.39–15.65), feeding support dependency (OR = 8.10, 95% CI: 6.27–10.48, p < 0.001), and urinary catheterization (OR = 4.08, 95% CI: 2.81–5.91, p < 0.001). In the multiple logistic regression analysis, the risk factors associated with aspiration pneumonia after propensity-adjustment (258 subjects each) were sputum suctioning (OR = 3.276, 95% CI: 1.910–5.619), deterioration of swallowing function in the past 3 months (OR = 3.584, 95% CI: 1.948–6.952), dehydration (OR = 8.019, 95% CI: 2.720–23.643), and dementia (OR = 1.618, 95% CI: 1.031–2.539).

**Conclusion:**

The risk factors for aspiration pneumonia were sputum suctioning, deterioration of swallowing function, dehydration, and dementia. These results could help improve clinical management for preventing repetitive aspiration pneumonia.

## Introduction

Pneumonia is a leading cause of hospitalisation and mortality among older adults, particularly those aged ≥85 years [1, 2]. Although aspiration pneumonia is the dominant form of community-acquired pneumonia (CAP) and healthcare-associated pneumonia (HCAP), the lack of specific and sensitive markers of aspiration complicates clinical and epidemiological investigations [3, 4]. Several studies have indicated that 7–24% of CAP is due to aspiration [5, 6]. We have previously reported that approximately 60% of hospitalized patients with CAP can be diagnosed with aspiration pneumonia [7]. Previous studies have shown that the risk of aspiration pneumonia increases with age, if patients are residing in a nursing home, and if a patient’s status is complicated with many geriatric disorders [8]. However, given that the average life span of Japanese women is 86 years, age itself may not be a risk factor for developing pneumonia.

There are many therapeutic guidelines for CAP and HCAP [9–11]. These guidelines apply to adult patients, but not specifically to older patients who present with high mortality due to aspiration pneumonia. HCAP in the United States is mostly characterized by infection with multidrug-resistant pathogens. However, HCAP and nursing and healthcare-associated pneumonia (NHCAP) in Spain and Japan are not related to multidrug-resistant pathogens, and result in worse clinical outcomes than CAP because of comorbidities related to dysphagia or aspiration [12, 13]. In addition, the rate of HCAP has increased with advancing age in developed countries and most of the cases can be diagnosed as aspiration pneumonia [14].

Aspiration pneumonia occurs in older adults with polypathology who have two or more chronic symptomatic diseases as well as various negative health and functional characteristics [15]. The increase in the ageing population worldwide means that the risk factors for developing aspiration pneumonia in older adults receiving nursing care need to be elucidated for the prevention and efficient clinical management of aspiration pneumonia. The aim of the present study was to examine the clinically significant risk factors associated with aspiration pneumonia among older adults receiving nursing care.

## Methods

### Study design, data sources, and study population

We carried out an observational and cross-sectional study, using secondary data from a nationwide questionnaire survey conducted by the Japanese Association of Geriatric Health Services Facilities (JAGHSF) in October 2010. JAGHSF has more than 3500 geriatric health services facilities (GHSFs) in its membership, which provide medical, nursing, and rehabilitation services for elderly under the long-term care insurance in Japan for people aged over 65 years.

The questionnaire survey was conducted at 3800 randomly selected GHSFs and associated health facilities receiving elderly care. The study subjects were aged 65 years and older, and lived in 1121 GHSFs at the time of observation. People aged less than 65 years were excluded from the survey. We collected data on general characteristics of subjects, the duration of years from admission, dysfunction and clinical supports, comorbidities, and deterioration of health condition on subjects in 3 months including standing, moving and walking, and swallowing function. Data on activities of daily living (ADL) were collected from the data of International Classification System of Functioning, Disability and Health (ICF) staging, the assessment method for ADL condition of patients, which was modified by the JAGHSF from the ICF, standardised by the World Health Organization (WHO) [16, 17]. Using ICF staging [18, 19], the level of ADL concerning mobility, walking, recognition (orientation, communication, and cognitive oriented function), eating (swallowing and the necessity of eating assistance), toileting, bathing, and dressing (oral care and washing) were collected.

Aspiration pneumonia was defined as an episode in the 3 months previous to the observation period, which was diagnosed by geriatricians in each GHSF according to the consensus by Japanese Study Group by the committee of Japan Respiratory Society on Aspiration Pulmonary Disease definition as pneumonia in a patient with a predisposition to aspiration because of dysphagia or swallowing disorders [20]. Since the swallowing function was not able to assess in all GHSF using the specialized examination tests, the presence of overt symptoms of dysphagia or the medical history of aspiration was determined as swallowing disorders in the patients. This method was allowed by the consensus of JRS [20]. Age was analysed as the continuous variable and as the stratified age groups with 65–74yrs., 75–89yrs, and ≥90yrs. The incidence of fever with caused by acute infectious diseases was determined by the fever of over 37.5°C with the test on bacterial culture. The dehydration was defined by the ratio of blood urea nitrogen (BUN) to serum creatinine was greater than twenty.

The subjects were divided into two groups: those with and those without aspiration pneumonia. We compared the factors relating to the incidence of aspiration pneumonia as the primary outcome. The purpose of the present study included finding the clinical risk factors for the incidence of aspiration pneumonia. We used a propensity scoring method to match age, sex and ADL in subjects with and without aspiration pneumonia to characterise the probability of being exposed to the given covariates.

The study was approved by the Institutional Review Boards of the University of Tsukuba. Written informed consent was obtained from the subjects or their relatives, and we obtained written permission to evaluate and report the survey data from the JAGHSF. This process was permitted by the Ethical Review Boards for this observational study using secondary data from a national survey by the JAGHSF. Investigators kept datasets in password-protected systems and presented data while maintaining the anonymity of the study participants.

### Statistical analysis

Data were reported as percentages for categorical variables and as median with interquartile range (IQR; 25–75%) for continuous variables. The characteristics of subjects with and without aspiration pneumonia were compared using the χ^2^ test or Fisher’s exact test for categorical variables and Mann–Whitney *U* or Kruskal–Wallis tests for continuous variables. Factors influencing aspiration pneumonia were determined using logistic regression analysis with independent variables if p was <0.05 and odds ratio (OR) >2.0 by univariate analysis, and a step-wise selection method was used to select variables and confirmed by the forced entry method. We carried out pairwise 1:1 propensity score matching, using nearest neighbour matching method, for reducing the effect of bias by unbalanced covariates and potential confounding [21,22]. The propensity score was estimated using multiple logistic regression analysis that included the independent variables of age, sex and ADL level to match subjects with and without aspiration pneumonia, using stepwise backward elimination procedures with p<0.10 and confirmed by the forced entry method. We conducted a logistic regression analysis on propensity-matched case–control subjects using independent variables if p was <0.05 and OR >2.0 by univariate analysis, and a stepwise selection method was used to select predictive variables for aspiration pneumonia.

Data were analysed using SPSS version 20 (IBM, Armonk, NY, USA). For all analyses, significance levels were two-tailed, and p<0.05 was considered statistically significant.

## Results

### General and clinical characteristics of subjects

A total of 9930 subjects from 1121 participating geriatric facilities throughout Japan were eligible for the present study. Two hundred and fifty-nine subjects (2.6%) had an episode of aspiration pneumonia in the 3 months prior to the observation period. The general characteristics, functional impairment status, clinical assistance requirements, and comorbidities of the subjects were compared between those with and without aspiration pneumonia (Table 1).

**Table 1:** General and clinical characteristics of study subjects.

Total No.: 9930		With AP	Without AP	P value	OR	95% CI
		N = 259 (2.6%)	N = 9671 (97.4)			
**Gender–**male, n (%)		99 (38.2)	2292 (23.7)	<0.001	1.99	1.54–2.57
**Age**–median (IQR)		86 (81–92)	86 (80–91)	0.201		
65–74		22 (8.5)	909 (9.4)	0.197		
75–89		148 (57.1)	5797 (59.9)			
≥90		89 (34.4)	2965 (30.7)			
**Number of years from admission–**y, median (IQR)	n = 9831	1 (1–2)	2 (1–3)	0.035		
**Dysfunction and clinical supports**						
Hemiplegia/diplegia	n = 9698	137 (54.8)	3070 (32.5)	<0.001	2.52	1.96–3.24
BPSD	n = 9610	155 (62.2)	5407 (57.8)	0.088	1.21	0.93–1.56
Brain dysfunction	n = 9622	163 (64.4)	3270 (34.9)	<0.001	3.38	2.60–4.39
Hearing dysfunction	n = 9673	66 (26.2)	1603 (16.9)	<0.001	1.75	1.32–2.33
Visual dysfunction	n = 9673	47 (18.7)	737 (7.7)	<0.001	2.75	1.98–3.81
Pseudobulbar palsy	n = 9632	140 (57.1)	1369 (14.6)	<0.001	7.81	6.03–10.12
Sputum Suctioning		102 (27.2)	351 (3.6)	<0.001	17.25	13.16–22.62
Daily oxygen therapy	n = 9801	12 (4.7)	57 (0.6)	<0.001	8.29	4.39–15.65
Nutrition support (nasogastric tube・gastrostomy)	n = 9815	111 (43.2)	820 (8.6)	<0.001	8.10	6.27–10.48
Cardiac pacemaker	n = 9788	4 (1.6)	160 (1.7)	1.000	0.93	0.34–2.54
Urinary Catheterisation	n = 9795	35 (13.8)	360 (3.8)	<0.001	4.08	2.81–5.91
**Clinical backgrounds**						
Cerebral haemorrhage		27 (10.4)	699 (7.2)	0.054	1.94	1.00–2.24
Multiple cerebral infarction		15 (5.8)	245 (2.5)	0.003	2.37	1.38–4.05
Chronic heart failure		3 (1.2)	122 (1.3)	1.000	0.92	0.29–2.90
Myocardial infarction		2 (0.8)	17(0.2)	0.087	4.42	1.02–19.23
Angina pectoris		1 (0.4)	35 (0.4)	0.614	1.07	0.15–7.82
Arrhythmia		1 (0.4)	13 (0.1)	0.309	2.88	0.38–22.10
Arteriosclerosis obliterans		0 (0.0)	15 (0.2)	0.729	-	-
Diabetes mellitus		11 (4.2)	402 (4.2)	1.000	1.02	0.56–1.89
Femoral neck fracture		14 (5.4)	765 (7.9)	0.159	0.67	0.39–1.15
Osteoporosis		2 (0.8)	133 (1.4)	0.453	0.56	0.14–2.27
COPD		2 (0.8)	19 (0.2)	0.103	3.95	0.92–17.06
Asthma		2 (0.8)	22 (0.2)	0.129	3.41	0.80–14.59
Renal failure						
Depression		5 (1.9)	192 (2.0)	1.000	0.97	0.40–2.38
Cancer		1 (0.4)	48 (0.5)	1.000	0.78	0.11–5.65
Hypertension (with no dementia)		4 (1.5)	210 (2.2)	0.535	0.71	0.26–1.92
Dementia with Lewy bodies		3 (1.2)	42 (0.4)	0.112	2.69	0.83–8.73
Dementia (other than Lewy bodies)		100 (38.6)	3122 (32.3)	0.037	1.319	1.024–1.700
Parkinson disease		10 (3.9)	248 (2.6)	0.229	1.53	0.80–2.91
Diseases on oral, salivary gland, jaw		0 (0.0)	39 (0.4)	0.627	-	-
Disease on stomach and oesophagus		4 (1.5)	152(1.6)	1.000	0.98	0.36–2.67
Disease on small/large intestine		1 (0.4)	42 (0.4)	1.000	0.89	0.12–6.48
Liver, gallbladder, pancreas disease		0 (0.0)	43(0.4)	0.630	-	-
Urinary tract infection		8 (3.1)	115 (1.2)	0.015	2.65	1.28–5.48
**Deterioration of condition on subjects in 3months**						
Standing condition	n = 8578	58 (23.7)	806 (9.7)	<0.001	2.90	2.14–3.92
Condition of moving and walking	n = 8512	55 (22.6)	803 (9.7)	<0.001	2.72	2.00–3.70
Swallowing function	n = 7504	75 (32.8)	412 (5.7)	<0.001	8.11	6.05–10.88
Fall・Trauma		16 (6.2)	961 (9.9)	0.056	0.60	0.36–0.99
Fracture		1 (0.4)	99 (1.0)	0.380	0.38	0.05–2.70
Fever with acute infectious diseases (other than AP)		73 (28.2)	1034 (10.7)	<0.001	3.28	2.48–4.33
Fall・Trauma		16 (6.2)	961 (9.9)	0.056	0.60	0.36–0.99
dehydration		46 (17.8)	244 (2.5)	<0.001	8.34	5.92–11.76

AP, aspiration pneumonia; BPSD, Behavioural Psychological Symptoms of Dementia; COPD, chronic obstructive pulmonary disease

OR, odds ratio; 95%CI, 95% of confidence interval

The age distribution of subjects was not normal. The median age was 86 years in both groups with no significant difference between the groups (p = 0.201). Over half of the subjects in the total sample were aged 75–89 years, and the analysis stratified by age group also showed no significant difference between those with and those without aspiration pneumonia (p = 0.197). There were significantly more male subjects with aspiration pneumonia than without (p < 0.001). The subjects in the group with aspiration pneumonia had a higher OR for sputum suctioning [OR = 17.5, 95% CI: 13.16–22.62], daily oxygen therapy (OR = 8.29, 95% CI: 4.39–15.65), feeding support (nasogastric tube or gastrostomy) dependency (OR = 8.10, 95% CI: 6.27–10.48), and urinary catheterization (OR = 4.08, 95% CI: 2.81–5.91). Regarding comorbidities, only dementia, urinary tract infection, and multiple cerebral infarction differed significantly between the subjects with and without aspiration pneumonia. The condition of subjects with aspiration pneumonia deteriorated more regarding standing, moving and walking, and swallowing compared to subjects without aspiration pneumonia (Table 1).

ADL were assessed using the International Classification of Functioning (ICF) staging [18,19], which measures mobility, walking, recognition (orientation, communication, and cognitive oriented function), eating (swallowing and the necessity of eating assistance), toileting, bathing, and dressing (oral care and washing). For all ADL except bathing, subjects with aspiration pneumonia tended to present with lower levels, while subjects without aspiration pneumonia presented with varying levels (Table 2).

**Table 2:** Level of ADL according to ICF staging.

Total No.: 9930		With AP	Without AP	P value
		N = 259 (2.6%)	N = 9671 (97.4)	
Level on General Activities	n = 9814			<0.001
1		148 (57.6)	1973 (20.6)	
2		50 (19.5)	1229 (12.9)	
3		22 (8.6)	1250 (13.1)	
4		27 (10.5)	2888 (30.2)	
5		10 (3.9)	2217 (23.2)	
Level on moving and Moving	n = 9804			<0.001
1		120 (46.7)	1900 (19.9)	
2		129 (50.2)	5454 (57.1)	
3		8 (3.1)	1878 (19.7)	
4		0 (0.0)	255 (2.7)	
5		0 (0.0)	60 (0.6)	
Cognitive function: Orientation level	n = 9778			<0.001
1		86 (33.5)	836 (8.8)	
2		75 (29.2)	2068 (21.7)	
3		56 (21.8)	2507 (26.3)	
4		21 (8.2)	17.3 (17.9)	
5		19 (7.4)	2407 (25.3)	
Cognitive function: Communication level	n = 9801			<0.001
1		95 (37.0)	1221 (12.8)	
2		90 (35.0)	2016 (21.1)	
3		28 (10.9)	2006 (21.0)	
4		27 (10.5)	2202 (23.1)	
5		17 (6.6)	2099 (22.0)	
Cognitive function: level of cognitive activities	n = 9771			<0.001
1		61 (23.9)	611 (6.4)	
2		132 (51.8)	3277 (34.4)	
3		31 (12.2)	1636 (17.2)	
4		18 (7.1)	1782 (18.7)	
5		13 (5.1)	2210 (23.2)	
Having meals: level of dysphagia	n = 9784			<0.001
1		107 (42.0)	734 (7.7)	
2		82 (32.2)	1292 (13.6)	
3		34 (13.3)	1467 (15.4)	
4		14 (5.5)	1645 (17.3)	
5		18 (7.1)	4391 (46.1)	
Level of toileting	n = 9766			<0.001
1		50 (19.5)	504 (5.3)	
2		164 (63.8)	3343 (35.1)	
3		33 (12.8)	2165 (22.7)	
4		5 (1.9)	1550 (16.3)	
5		5 (1.9)	1960 (20.6)	
Level of bathing	n = 9746			<0.001
1		5 (2.0)	131 (1.4)	
2		199 (78.0)	3625 (38.2)	
3		48 (18.8)	4889 (51.5)	
4		2 (0.8)	579 (6.1)	
5		1 (0.4)	267 (2.8)	
Level of self-care: oral care	n = 9748			<0.001
1		178 (69.5)	2009 (21.2)	
2		44 (17.2)	1985 (20.9)	
3		20 (7.8)	2583 (27.2)	
4		4 (1.6)	970 (10.2)	
5		10 (3.9)	1945 (20.5)	
Level of self-care: level of dressing	n = 9748			<0.001
1		190 (74.2)	2673 (28.2)	
2		43 (16.8)	2288 (24.1)	
3		13 (5.1)	1664 (17.5)	
4		7 (2.7)	1623 (17.1)	
5		3 (1.2)	1244 (13.1)	

ICF staging was based on the International Classification System of Functioning, Disability and Health by the WHO [18, 19]. Each item was scored from 1 to 5, with decreasing score values (lower digit items presented difficult conditions).

### Risk factors for aspiration pneumonia among elderly subjects using multiple logistic regression analysis

The multiple logistic regression analysis adjusted for baseline factors indicated that male sex, sputum suctioning, daily oxygen therapy, feeding support dependency, urinary catheterization, deterioration of swallowing function in the past 3 months, fever with acute infectious disease, dehydration, and dementia were related to the incidence of aspiration pneumonia (Table 3).

**Table 3:** Factors influencing aspiration pneumonia by logistic regression analysis.

	Coefficient	SE	P value	Odds ratio	95% CI
Constant	−14.567	1.422			
Gender	0.537	0.171	0.002	1.710	1.223–2.391
Sputum suctions	1.499	0.221	<0.001	4.477	2.901–6.909
Daily oxygen therapy	1.744	0.560	0.002	5.719	1.908–17.145
Nutrition support (nasogastric tube or gastrostomy)	1.213	0.210	<0.001	3.362	2.227–5.077
Urinary Catheterisation	0.615	0.255	0.016	1.850	1.122–3.052
Deterioration of swallowing function	1.565	0.188	<0.001	4.783	3.310–6.911
Fever with acute infectious diseases (other than aspiration pneumonia)	0.703	0.183	<0.001	2.020	1.410–2.894
Dehydration	1.426	0.244	<0.001	4.163	2.583–6.711
Dementia	0.435	0.164	0.008	1.545	1.121–2.129

SE, standard error; 95%CI, 95% of confidence interval

In addition, a propensity-score-matched case-control study was conducted among a subsample of 514 subjects with and without aspiration pneumonia. The logistic regression analysis showed that sputum suctioning, deterioration of swallowing function in the past 3 months, dehydration, and dementia were associated with the incidence of aspiration pneumonia (Table 4).

**Table 4:** Factors influencing aspiration pneumonia using logistic regression analysis in propensity-matched case–control study.

	Coefficient	SE	P value	Odds ratio	95% confidence interval
Constant	−9.347	1.386			
Sputum suctioning	1.187	0.275	<0.001	3.276	1.910–5.619
Deterioration of swallowing function	1.276	0.311	<0.001	3.584	1.948–6.592
Dehydration	2.082	0.552	<0.001	8.019	2.720–23.643
Dementia	0.481	0.230	0.036	1.618	1.031–2.539

SE, standard error; OR, odds ratio; 95%CI, 95% of confidence interval

## Discussion

The present comprehensive study identified that the predictive clinical factors for developing aspiration pneumonia in older adults receiving nursing care were sputum suctioning, deterioration of swallowing function in the past 3 months, dehydration, and dementia. These findings could improve the clinical management and prevention of repetitive aspiration pneumonia, a leading cause of death in older adults.

One hundred years ago, Sir William Osler said that pneumonia is a friend of the aged. Indeed, elderly people are considerably more likely to suffer and die from pneumonia, in particular aspiration pneumonia [23]. Therefore, it is necessary to determine the predictive factors for aspiration pneumonia in the high-risk subgroup of elderly people receiving nursing care. Importantly, whereas the majority of previous studies enrolled subjects aged 50–75 years, the median age of our study subjects was 86 years providing a representative age sample for aspiration pneumonia [24–27].

Japan is an ageing country and, in this study, we were able to determine the significant risk factors for aspiration pneumonia in an elderly Japanese population. In accordance with previous studies [24–27], the multiple regression analysis revealed several risk factors for aspiration pneumonia (Table 3). However, the propensity-adjusted analysis found only four major clinical risk factors (Table 4). In previous studies examining the features of aspiration pneumonia, the analyzed elderly age group was younger than those of the present study. This study, on the other hand, revealed the significant risk factors for aspiration pneumonia among frail, older elderly people and found that age did not present as a significant risk factor among the older elderly in the present study.

Although patients who required intensive and advanced care were not in a geriatric health service facility (GHSF), our study subjects included a variety of elderly people with polypathology [15] who were receiving standard medical care along with nursing care. The comorbidity of our subjects varied (Table 1). A recent study on people aged <80 years identified chronic obstructive pulmonary disease (COPD) as a risk factor for aspiration pneumonia [24]. However, the present study indicated that comorbid diseases (except multiple cerebral infarction) did not affect the incidence of aspiration pneumonia (Table 1). Although there was no detailed examination of ADL in the previous study [25], a decrease in ADL is thought to be a risk factor for aspiration pneumonia. The present study revealed that decreased standing, moving and walking ability, as well as impaired swallowing function were observed significantly more often in subjects with aspiration pneumonia than in those without aspiration pneumonia (Table 2).

We further evaluated the data for the total sample using a multiple logistic regression analysis to establish the factors that influence the incidence of aspiration pneumonia. These factors were male sex, sputum suctioning, daily oxygen therapy, nutrition support dependency (nasogastric tube or gastrostomy), urinary catheterization, deterioration of swallowing function in the past 3 months, fever with acute infectious disease, dehydration, and dementia (Table 3). However, these variables are potentially interdependent with health status indicators and disease conditions; therefore, we also examined the clinically significant risk factors associated with the development of aspiration pneumonia among elderly, propensity-matched subjects receiving nursing care. Although propensity score matching has several limitations [21,22], we conducted a multiple regression analysis among the propensity-adjusted subjects to reduce the imbalance between those with and without aspiration pneumonia, especially for ADL levels. This analysis was important for investigating the clinical risk factors in an older elderly population receiving nursing care and found that sputum suctioning, dysphagia, dehydration, and dementia were significant risk factors for recurrent aspiration pneumonia in this population (Table 4). People living in GHSFs receive standard care from qualified staff, thus, sputum suctioning indicates impairment in saliva swallowing and mis-swallowing of airway secretions among patients. This particular risk factor may not have been recognized in previous studies in community-dwelling settings given the limited sputum suctioning. Furthermore, family caregivers often have difficulty recognizing the possible causes of aspiration pneumonia. However, recent research has suggested that impairment of saliva swallowing and mis-swallowing of airway secretions are major causes of aspiration pneumonia in elderly people [26]. These risk factors indicate the need for conventional swallowing assessments to determine dysphagia [27,28]. However, conventional swallowing assessments focus on feeding ability and not mis-swallowing of saliva and airway secretions (except during meals). In addition to meal dysphagia, mis-swallowing of saliva and airway secretions during the night are significant risk factors for developing aspiration pneumonia [29–32].

In this study, dehydration was shown to be an important risk factor for aspiration pneumonia. Although dehydration is a known risk factor for pneumonia, previous studies have not indicated that it is a risk factor for aspiration pneumonia in particular. The elderly subjects in this study received standard care in their facilities as well as rehabilitation services. In this setting, dehydration indicates impairment of oral intake and feeding. Thus, dehydration may be a good indicator of nutrition status in elderly people.

Our study also showed that dementia is a risk factor for the development of aspiration pneumonia. Studies have recently reported that elderly dementia patients (mean age of 86 years) inevitably develop dysphagia and have a high risk of aspiration pneumonia and related mortality in the hospital [33]. Even in special facilities that provide total care for elderly people, dementia may not be totally controlled by clinical staff. Therefore, it is reasonable to assume that dementia is a significant risk factor for aspiration pneumonia.

These results are important for elderly people receiving nursing care. Several risk factors can cause repetitive and recurrent aspiration pneumonia especially in older people, and the mortality of recurrent aspiration pneumonia is high in older adults [14,34]. A discussion regarding the feasibility of intensive therapy with or without mechanical ventilation as well as prior selection of antibiotics is needed. In light of this, the Japanese NHCAP guidelines discuss the feasibility of intensive therapy of aspiration pneumonia.

The present study had some limitations. Although aspiration pneumonia in the present study was clinically diagnosed based on the consensus by the Japanese Respiratory Society (JRS) committee of the Japanese Study Group on Aspiration Pulmonary Diseases [20], we were not able to assess swallowing function using functional assessments (e.g., the water swallowing test, repetitive saliva swallowing test, or simple-swallowing provocation test) for all patients residing in a GHSF. However, the presence of overt symptoms of dysphagia and a medical history of aspiration were considered as swallowing disorders in the patients. This method was approved by consensus from the JRS. The observational period for assessing the incidence of aspiration pneumonia was 3 months; however, the incidence of aspiration pneumonia might increase if the observation period were extended. These limitations suggest the necessity of a universal consensus for the aspiration pneumonia diagnostic criteria and additional investigations for evaluating the risk factors of aspiration pneumonia in a rapidly ageing population.

## Conclusion

Our comprehensive, observational study established that the predictive clinical risk factors for aspiration pneumonia in older adults receiving nursing care were: sputum suctioning, deterioration of swallowing function, dehydration, and dementia. The results could be incorporated into clinical practice to prevent and reduce repetitive aspiration pneumonia. The results warrant further investigation in a prospective cohort study.

## References

[1] Kaplan V, Angus DC, Griffin MF, Clermont G, Scott Watson R, Linde-Zwirble WT (2002) Hospitalized community-acquired pneumonia in the elderly: age- and sex-related patterns of care and outcome in the United States. Am J Respir Crit Care Med 165(6):766–72. 11897642 10.1164/ajrccm.165.6.2103038

[2] Loeb M, McGeer A, McArthur M, Walter S, Simor AE (1999) Risk factors for pneumonia and other lower respiratory tract infections in elderly residents of long-term care facilities. Arch Intern Med 159(17):2058–64. 10510992 10.1001/archinte.159.17.2058

[3] Marik PE, Kaplan D (2003) Aspiration pneumonia and dysphagia in the elderly. Chest 124(1):328–336. 12853541 10.1378/chest.124.1.328

[4] Reza Shariatzadeh M, Huang JQ, Marrie TJ (2006) Differences in the features of Aspiration Pneumonia According to Site of Acquisition: Community or Continuing Care Facility. J Am Geriatr Soc 54(2):296–302. 16460382 10.1111/j.1532-5415.2005.00608.x

[5] Marrie TJ (1990) Epidemiology of community-aquired pneumonia in the elderly. Semin Respir Infect 5(4):260–8. 2093971

[6] Leroy O, Vandenbussche C, Coffinier C, Bosquet C, Georges H, Guery B, et al (1997) Community-acquired aspiration pneumonia in intensive care units. Epidemiological and prognosis data. 156(6):1922–9. 10.1164/ajrccm.156.6.9702069 9412576

[7] Teramoto S, Fukuchi Y, Sasaki H, Sato K, Sekizawa K, Matsuse T; et al (2008) High incidence of aspiration pneumonia in community- and hospital-acquired pneumonia in hospitalized patients: a multicenter, prospective study in Japan. J Am Geriatr Soc 56(3):577–9. 10.1111/j.1532-5415.2008.01597.x 18315680

[8] Muder RR (1998) Pneumonia in residents of long-term care facilities: epidemiology, etiology, management, and prevention. Am J Med 105(4):319–30. 9809694 10.1016/s0002-9343(98)00262-9

[9] American Thoracic Society and the Infectious Diseases Society of America (2005) Guidelines for the management of adults with hospital-acquired, ventilator-associated, and healthcare-associated pneumonia. Am J Respir Crit Care Med 171:388–416. 15699079 10.1164/rccm.200405-644ST

[10] The committee for the Japanese Respiratory Society guidelines in management of respiratory infections (2006) The Japanese Respiratory Society guideline for the management of community-acquired pneumonia in adults. Respirology 11: S1–S133.16423260

[11] The committee for the Japanese Respiratory Society guidelines in management of respiratory infections (2009) The Japanese Respiratory Society guideline for the management of hospital-acquired pneumonia in adults 2008. Respirology 14: S1–S71.19857216

[12] Polverino E, Torres A, Menendez R, Cilloniz C, Valles JM, Capelastegui A, et al (2013) Microbial aetiology of healthcare associated pneumonia in Spain: a prospective, multicentre, case-control study. Thorax 68(11):1007–14. 10.1136/thoraxjnl-2013-203828 24130227

[13] Fukuyama H, Yamashiro S, Tamaki H, Kishaba T (2013) A prospective comparison of nursing- and healthcare-associated pneumonia (NHCAP) with community-acquired pneumonia (CAP). J Infect Chemother 19(4):719–26. 10.1007/s10156-013-0557-1 23354936

[14] Teramoto S, Kawashima M, Komiya K, Shoji S (2009) Health-care-associated pneumonia is primarily due to aspiration pneumonia. Chest. 136(6):1702–3; 10.1378/chest.09-1204 19995780

[15] Jadad AR, Cabrera A, Martos F, Smith R, Lyons RF (2010) When people live with multiple chronic diseases: a collaborative approach to an emerging global challenge Seville: Andalusian Government; 2010. Available: http://www.opimec.org/equipos/when-people-live-with-multiple-chronic-diseases/

[16] An J, Shim JH, Kim SO, Lee D, Kim KM, Lim YS, et al (2014) Prevalence and Prediction of Coronary Artery Disease in Patients with Liver Cirrhosis: A Registry-Based Matched Case Control Study. Circulation 130(16):1353–62. 10.1161/CIRCULATIONAHA.114.009278 25095888

[17] Kim KH, Kim YM, Kim MC, Jung GJ (2014) Analysis of prognostic factors and outcomes of gastric cancer in younger patients: A case control study using propensity score methods. World J Gastroenterol 20(12):3369–75. 10.3748/wjg.v20.i12.3369 24696617 PMC3964409

[18] World Health Organization (2001) International classification of functioning, disability and health: ICF Geneva World Health Organization Available http://psychiatr.ru/download/1313?view=1&name=ICF_18.pdf.

[19] Okochi J, Utsunomiya S, Takahashi T (2005) Health measurement using the ICF: test-retest reliability of ICF codes and qualifiers in geriatric care. Health and Quality of Life Outcomes 3:46 16050960 10.1186/1477-7525-3-46 PMC1199614

[20] Japanese Respiratory Society. Aspiration pneumonia. Respirology. 2009;14 Suppl 2:S59–64. 10.1111/j.1440-1843.2009.01578.x 19857224

[21] Rosenbaum PR, Rubin DB (1983) The central role of the propensity score in observational studies for causal effects. Biometrika 70:41–55.

[22] Rezaeian S, Poorolajal J, Moghimbegi A, Esmailnasab N (2013) Risk factors of congenital hypothyroidism using propensity score: a matched case-control study. J Res Health Sci 13(2):151–6. 24077472

[23] Osler W (1998) The Principles and Practice of medicine New York: D. Appleton and Co: 109.

[24] Cameron JL, Mitchell WH, Zuidema GD (1973) Aspiration pneumonia. Clinical outcome following documented aspiration. Arch Surg 106(1):49–52. 4565234 10.1001/archsurg.1973.01350130051011

[25] Hibberd J, Fraser J, Chapman C, McQueen H, Wilson A (2013) Can we use influencing factors to predict aspiration pneumonia in the United Kingdom?. Multidisciplinary Respiratory Medicine 8:39 10.1186/2049-6958-8-39 23758693 PMC3686575

[26] Taylor JK, Fleming GB, Singanayagam A, Hill AT, Chalmers JD. Risk factors for aspiration in community-acquired pneumonia: analysis of a hospitalized UK cohort. Am J Med. 2013;126(11):995–1001. 10.1016/j.amjmed.2013.07.012 24054176

[27] Hu X, Yi ES, Ryu JH (2014) Aspiration-Related Deaths in 57 Consecutive Patients: Autopsy Study. PLoS ONE 9(7): e103795 10.1371/journal.pone.0103795 25076409 PMC4116222

[28] Langmore SE, Terpenning MS, Schork A, Chen Y, Murray JT, Lopatin D, et al (1998) Predictors of aspiration pneumonia: How important is dysphagia? Dysphagia 13:69–81. 9513300 10.1007/PL00009559

[29] Teramoto S, Fukuchi Y (2000) Detection of aspiration and swallowing disorder in older stroke patients: simple swallowing provocation test versus water swallowing test. Arch Phys Med Regabil 81(11):1517–9. 10.1053/apmr.2000.9171 11083358

[30] Teramoto S, Matsuse T, Fukuchi Y, Ouchi Y (1999) Simple two-step swallowing provocation test for elderly patients with aspiration pneumonia. Lancet 353(9160):1243 10217091 10.1016/S0140-6736(98)05844-9

[31] Teramoto S, Yamamoto H, Yamaguchi Y, Ouchi Y, Matsuse T (2004) A novel diagnostic test for the risk of aspiration pneumonia in the elderly. Chest 125(2):801–2. 14769774 10.1378/chest.125.2.801

[32] Kikuchi R, Watabe N, Konno T, Mishina N, Sekizawa K, Sasaki H (1994) High incidence of silent aspiration in elderly patients with community-acquired pneumonia. Am J Respir Crit Care Med 150(1):251–3. 8025758 10.1164/ajrccm.150.1.8025758

[33] Bosch X, Formiga F, Cuerpo S, Torres B, Rosón B, López-Soto A (2012) Aspiration pneumonia in old patients with dementia. Prognostic factors of mortality. Eur J Intern Med 23(8):720–6. 10.1016/j.ejim.2012.08.006 22964260

[34] Hayashi M, Iwasaki T, Yamazaki Y, Takayasu H, Tateno H, Tazawa S, et al (2014) Clinical features and outcomes of aspiration pneumonia compared with non-aspiration pneumonia: a retrospective cohort study. J Infect Chemother 20(7):436–42. 10.1016/j.jiac.2014.04.002 24834866

